# Effect of medical innovation policies on the prevention and control of the COVID-19 and the impact of the “Belt and Road” economy

**DOI:** 10.3389/fpubh.2022.862487

**Published:** 2022-08-29

**Authors:** Yirun Chen, Zichao Zhao, Wensheng Dai

**Affiliations:** Financial School, China Financial Policy Research Center, International Money Institute, Renmin University of China, Beijing, China

**Keywords:** medical innovation policy, new crown epidemic prevention and control, “One Belt, One Road” economy, convergence model, epidemic

## Abstract

With the spread of the COVID-19, it is urgent for everyone to protect themselves. The introduction of the medical innovation policy has also brought certain effects to the prevention and control of the COVID-19. The specific effect will be reflected in the following research. This paper firstly analyzed research results related to medical innovation policy, COVID-19 prevention and control, and the “One Belt, One Road” economy, finding out the content that fits this research, and innovates the research work on this basis. Then, this paper provided a detailed explanation of medical innovation policies, the prevention and control of the COVID-19, and the “One Belt, One Road” economy. Among them, this paper focuses on the “One Belt and One Road,” uses the α-convergence model to analyze the economic changes of the “One Belt and One Road,” and conducts experimental tests in the medical field. The results have shown that from 2017 to 2019, the average hospitalization expenses paid by the pooled funds were 4986.19, 4997.34, and 4888.60 yuan, respectively.

## Introduction

With the construction of the “Belt and Road,” the initiative has gradually turned into practice, from theory to action. Some Western scholars and media have begun to play up a tone, believing that China's move is to manage its own sphere of influence and strive for regional dominance. Therefore, they regard China's “Belt and Road” initiative as the Marshall Plan during the Cold War, and even mistake it as China's political and security strategy under the shifting of global power. The purpose of this paper is to explore the relationship between the economic development of the countries along the “Belt and Road” cooperation mechanism that promotes each other rather than competes with each other. From the empirical results, it can be seen that the economic development of neighboring countries has a mutually promoting relationship in space. Moreover, the rapid economic growth of a country can speed up the convergence of neighboring countries to their own steady state. That is to say, although the technological progress of a country makes the relative gap between neighboring countries tend to widen, due to technological spillover, the absolute growth rate of neighboring countries is improved, and the convergence to its own steady state is accelerated.

However, the Belt and Road Initiative aims at common development and common prosperity, and is characterized by inclusiveness and openness. First of all, today's world economy is increasingly interconnected, and the rapid growth and sustainable development of a country's economy must depend on the healthy economic development of other countries, which is completely different from the era background of the Marshall Plan. Secondly, most of the countries along the “Belt and Road” are developing countries, all of which have common problems of lack of funds and lagging technology, and they are all in the relatively lagging stage of urbanization and industrialization. Experience since World War II shows that developing countries that can develop labor-intensive industries can achieve rapid economic growth for about 30 years. The “One Road, One Belt” cooperation mechanism strengthens interconnection, trade flow, factor flow, and technology flow. It can enable relatively backward countries to share information and services of neighboring manufacturing and technologically advanced countries. It is also conducive to expanding the market size and achieving economies of scale. More importantly, due to the disappearance of China's demographic dividend, the comparative advantage of labor-intensive industries is gradually lost. By improving infrastructure construction, countries along the route can better absorb labor-intensive industries transferred from China. This huge process of labor absorption can not only promote employment in countries along the route, but also increase per capita income, expand consumption potential, and improve people's welfare.

The main innovations of this paper are:

1. This paper adopts the spatial panel data analysis method to overcome the problems of variable omission and endogeneity in the cross-sectional data analysis method. At the same time, after the introduction of spatial effects, the interaction of countries along the route is included in the model for analysis, and each country is no longer regarded as independent and unrelated economies. Therefore, it can better demonstrate that the “Belt and Road” initiative is about strengthening infrastructure construction, improving trade facilitation and enhancing factor flow, and trade exchanges can further strengthen the economic connections and spatial effects of countries along the route.
2. From the perspective of the integration of urban and rural residents' medical insurance, this paper quantitatively analyzes the changes in medical expenses before and after the integration of urban and rural residents' medical insurance. At present, most researches on medical expenses at home and abroad focus on one aspect of medical expenses. In addition, most domestic researches on medical insurance for urban and rural residents remain on system design, path selection and the necessity of integrating urban and rural medical insurance. It lacks the empirical analysis of related medical expenses after the integration of urban and rural medical insurance. Based on the background of the integration of urban and rural residents' medical insurance, this paper studies the current medical insurance cost control issues. It uses breakpoint regression to analyze the impact of urban and rural residents' medical insurance merger on medical expenses, which is innovative to a certain extent.
3. In the empirical analysis of this paper, due to the large amount of individual medical insurance data and information in a certain area, there is heterogeneity among individuals, and the information on individual characteristics is not very complete and accurate. In the model estimation, it may lead to ignore the role of some influence paths, and finally affect the model estimation result.
4. This study provides a detailed description of telemedicine in medical innovation policy, thus reflecting the benefits brought by medical innovation policy to society.

## Related work

Many scholars have provided a lot of references for research on medical innovation policies, COVID-19 prevention and control, and the “Belt and Road” economy.

Moreira et al. analyzed whether several types of innovation affect relevant measures of medical institution performance, and empirically studies the relationship between innovation and performance. Based on a quantitative analysis of 34 Portuguese hospitals, the study gathered detailed information on each hospital's innovation portfolio. In addition, he classified hospitals by attribute type and geographic region, and performed statistical comparison tests to check for statistical differences. The study found that organizational innovation is related to process innovation and service innovation. In addition, service and process innovations impact operational performance. However, Moreira et al. cannot conclude that innovations in healthcare units have an overall impact on their financial performance (1).

Hossein proposed a fast online identification scheme to identify suitable linear state-space models for use in control algorithms. Then, he proved the convergence of recognition error sequences using Lyapunov candidate functions in both time and frequency domains (graph topology) (2).

Garmann-Johnsen et al. explored and conceptualizes how Nordic principles of employee engagement combined with corporate social media/web 2.0 can enable co-creation as an input in the digital transformation of healthcare delivery. Garmann-Johnsen et al. first introduced the Nordic model of employee engagement, which has proven successful in improving work-life efficiency and innovation. Garmann-Johnsen et al. then discussed how these engagement principles can be further enhanced by the latest web 2.0 technologies of enterprise social networking. To extend this further, Garmann-Johnsen et al. uses a practical template to exemplify the co-design of therapeutic pathway strategies (3).

Alam et al. aimed to explore the current state of digital transformation of the healthcare service sector in public, private and NGOs, as well as the management and technical challenges facing the digitisation of healthcare projects in Bangladesh. Research has shown that although there are still some problems with the digitization of the sector, difficulties and challenges can be overcome. According to the assessment of this sector, the scope of some areas needs to be further improved. These findings will help government agencies, policymakers, healthcare providers, and mobile phone companies make effective decisions about the digitization of healthcare services (4).

Proksch et al. compared the healthcare innovation outputs in 30 OECD countries using a multi-indicator approach and used cluster analysis to divide them into four distinct groups. The cluster consisting of the Scandinavian countries, the Netherlands and Switzerland showed the highest innovation output in terms of knowledge production and knowledge commercialization (5).

Shoemaker et al. supports the establishment of cross-jurisdictional approaches to facilitate streamlined data collection and uninterrupted connections and allow for meaningful analysis to inform national policy (6).

Lehoux et al. illuminated how entrepreneurs, investors and regulators can influence the value of emerging health technologies. The 5-year qualitative research program examines the process by which entrepreneurial clinical teams operating in Canada's publicly funded healthcare system envision, fund, develop and commercialize new health technologies. The results have suggested that policy-oriented initiatives, such as early health technology assessment (HTA) and evidence coverage, can provide technology developers with useful information about their early decision-making. But to foster technologies that bring more value to the healthcare system, policymakers must actively support the consideration of health policy issues in innovation policy (7).

The data of these studies are not comprehensive, and the results of the studies are still open to question, so they cannot be recognized by the public and thus cannot be popularized and applied.

## Medical innovation policies, COVID-19 prevention and control and the “Belt and Road” economy

### Medical innovation policy

**Figure 1:**
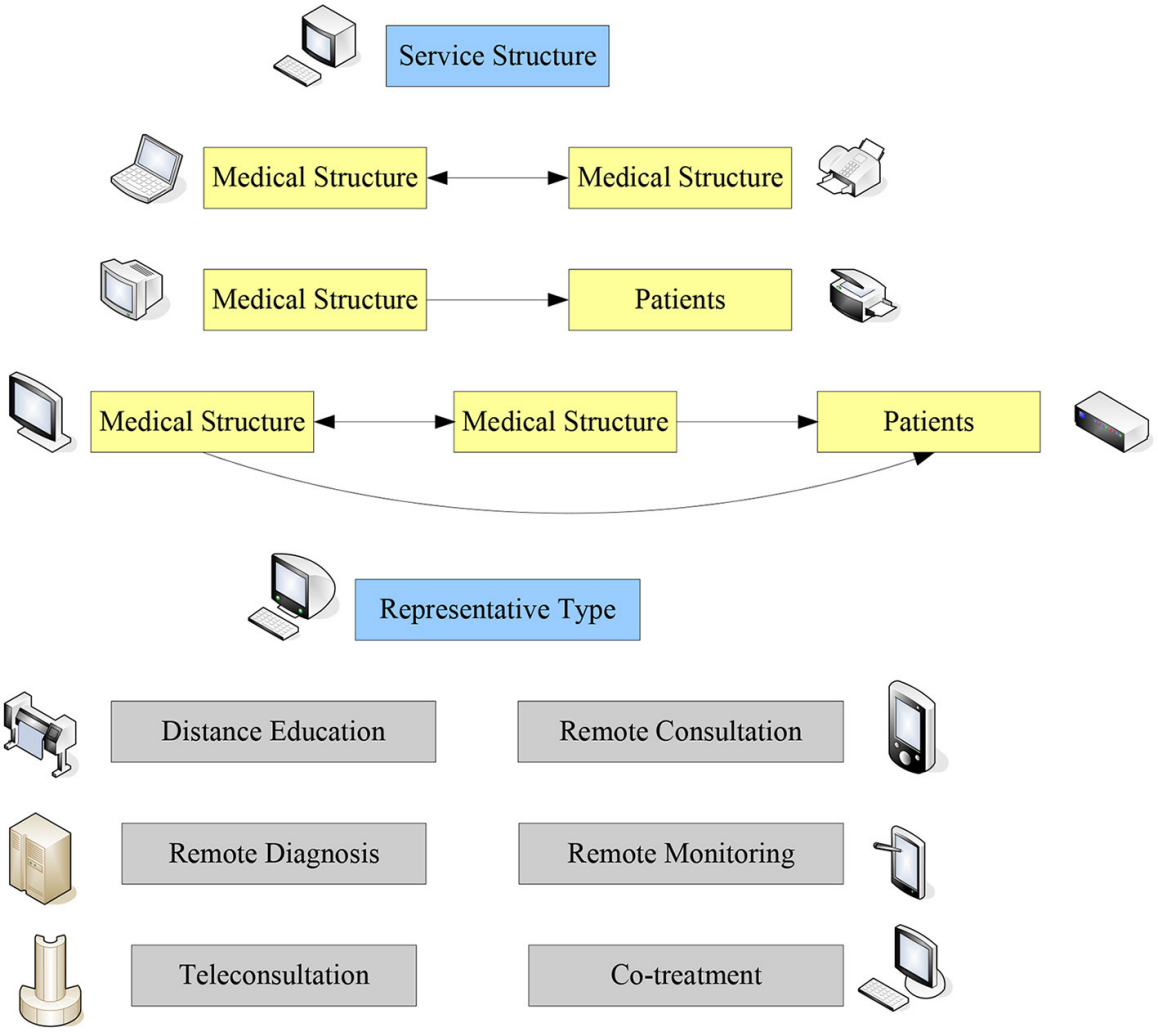
Content classification of telemedicine.

The medical innovation policy is a reform of the existing medical policy, and some of the existing medical policies have some drawbacks. It not only consumes a lot of manpower and material resources, but also fails to achieve the expected results, so there is the emergence of medical innovation policies. Telemedicine is a very promising technology, and the description of this medical innovation policy will focus on telemedicine. In terms of content, telemedicine can be expressed as remote diagnosis, remote consultation, remote education, remote monitoring and remote treatment. From the service structure, the content of telemedicine services can be divided into three types, as shown in Figure 1. The first is the discussion, consultation or online medical education between medical institutions and medical institutions that does not involve the personal information of patients. The second is the direct remote diagnosis of patients by medical institutions, or the monitoring and management of chronic diseases through wearable devices and monitoring equipment. The third is to include medical activities between remote medical institutions and proximal medical institutions and patients, such as remote consultation, remote joint diagnosis and treatment, and remote surgery (8, 9). The so-called telemedicine should be understood as telemedicine in a broad sense, that is, telemedicine activities including tele-information transmission and tele-education (10).

**Figure 2:**
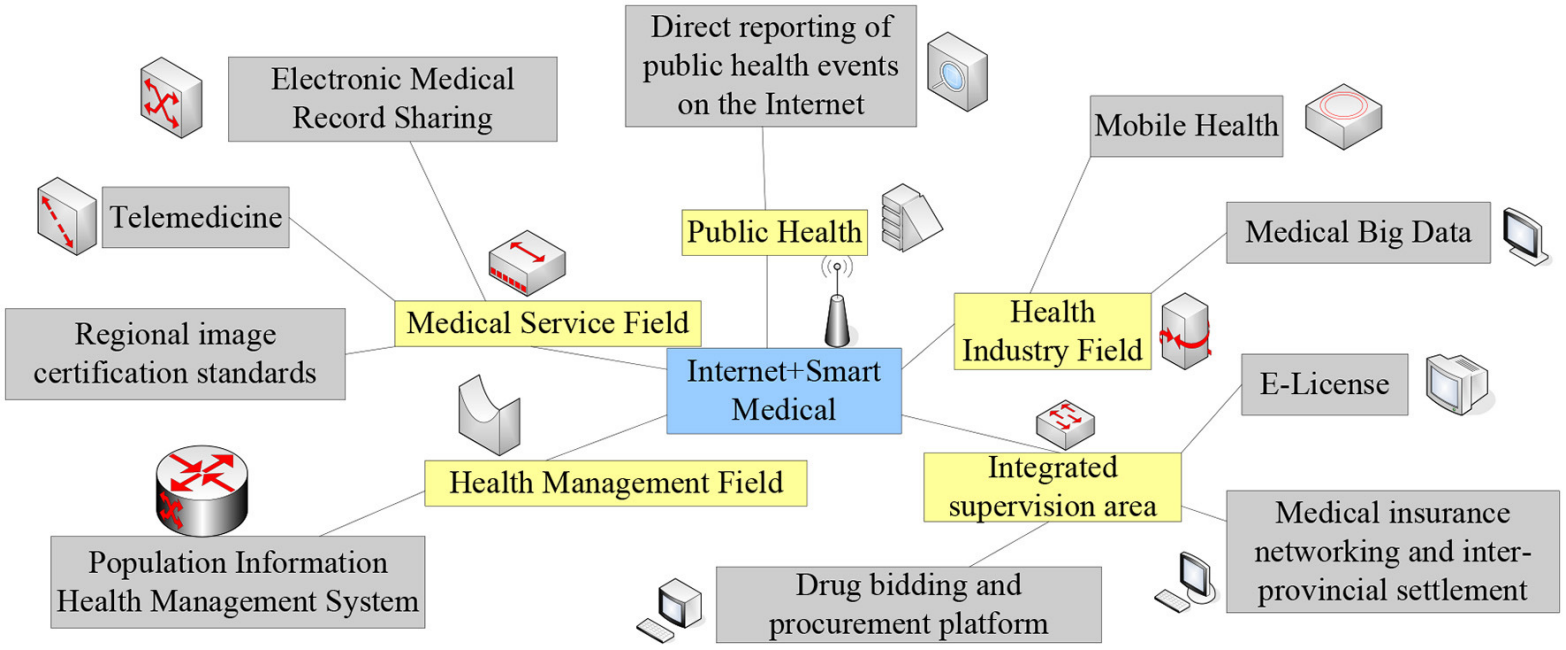
“Internet + Smart Medical” system structure.

The realization of today's telemedicine depends on the cooperation of many factors, but the importance of the network is self-evident. The birth of 5G has brought great changes to telemedicine, and the real-time information transmission of the network will enable many operations of telemedicine to be implemented (11, 12). The architecture diagram of “Internet + Smart Healthcare” is shown in Figure 2.

**Figure 3:**
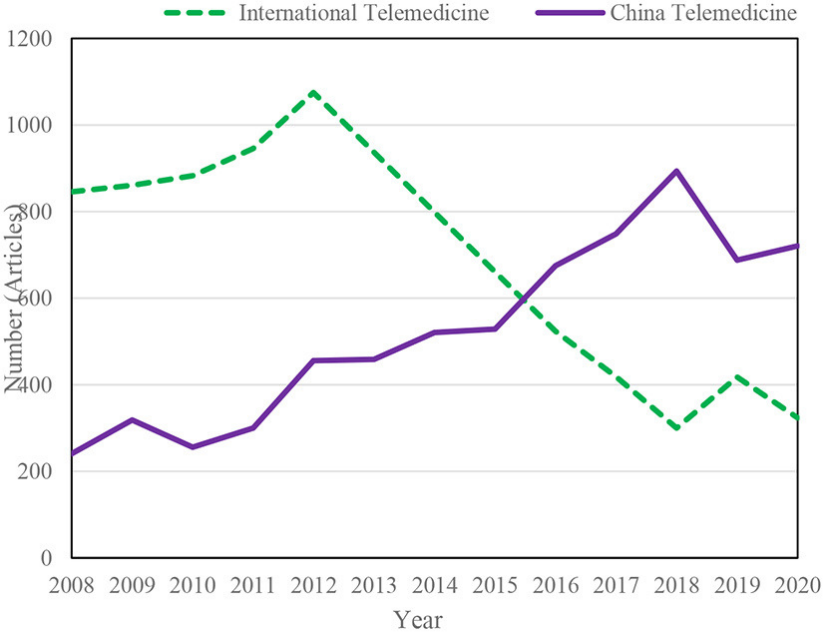
Research trends in Chinese and international telemedicine literature, 2008–2020.

**Figure 4:**
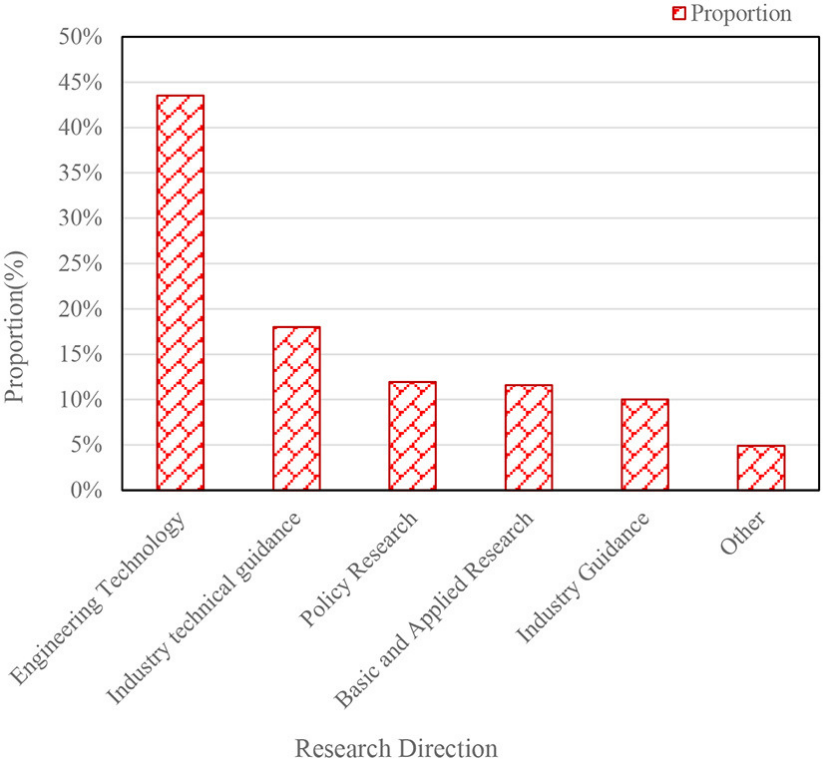
Research directions in Chinese telemedicine literature.

The research trends of Chinese and international telemedicine literature from 2008 to 2020 are shown in Figure 3 (13, 14). The research direction of Chinese telemedicine literature is shown in Figure 4 (15, 16).

### Prevention and control of the COVID-19

The clinical manifestations of new coronavirus pneumonia include: sudden onset, chills and high fever, body temperature up to 39–40°C, and many systemic symptoms such as headache, general muscle and joint pain, extreme fatigue, and loss of appetite. It often has sore throat, dry cough, nasal congestion, runny nose, and retrosternal discomfort.

**Figure 5:**
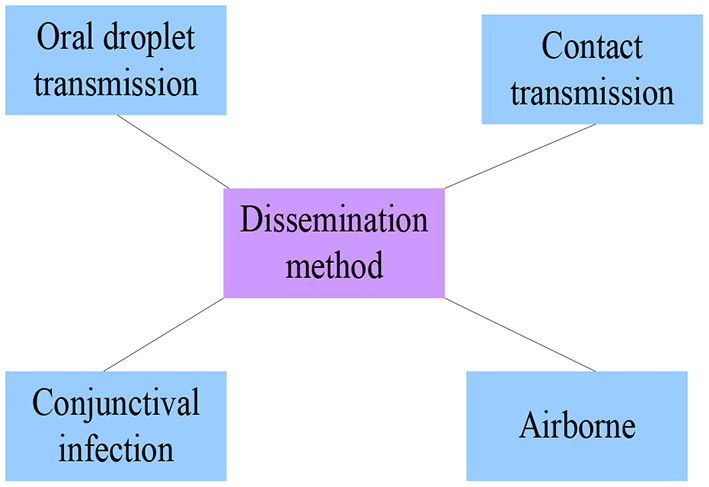
How the new crown outbreak spreads.

The COVID-19 is now spreading all over the world, but it has been contained to a certain extent with the efforts of people. What people need to take seriously now is how to protect themselves. In order to do a good job in the prevention and control of the COVID-19, it is necessary to understand the transmission mode of the COVID-19 from the source, as shown in Figure 5 (17, 18).

**Figure 6:**
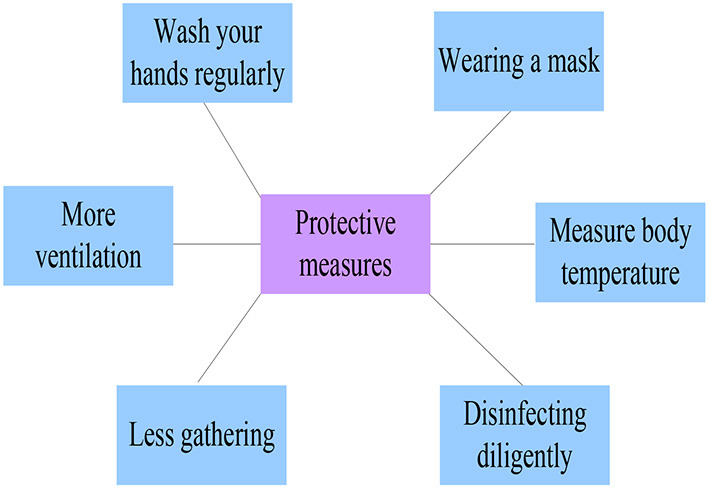
Protective measures for the new crown outbreak.

After understanding the transmission mode of the COVID-19, a series of protective measures starting from blocking the transmission can be formulated. The specific protective measures are shown in Figure 6 (19, 20).

### “Belt and Road” economy

**Figure 7:**
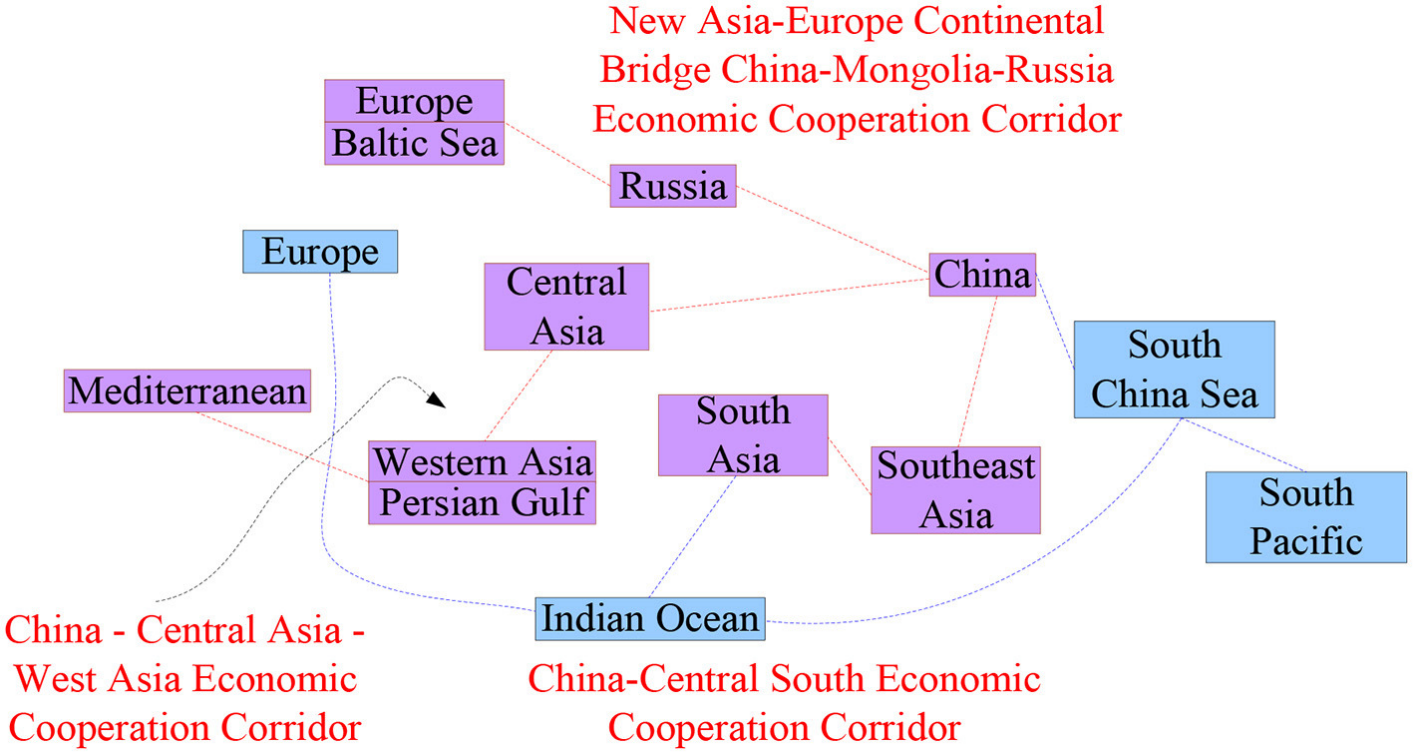
The route of “One Belt, One Road”.

The “Belt and Road” construction is not only for China to deal with its own development problems, but more importantly, it injects new impetus into the world economic growth after the financial crisis (21, 22). Under the pressure of the overall weakness of the world economy and the structural contradictions of its own development, China's economy will enter a state of medium-high growth. In order to maintain the steady growth of China's economy, it has overcome difficulties from home and abroad (23, 24). The route of the “Belt and Road” is shown in Figure 7.

Before the “One Belt, One Road” initiative was proposed, many people believed that the Silk Road was a small intestine path that could only pass camels and horses, and could only transport light materials such as spices, porcelain and silk. In fact, the Silk Road was formed by the diffusion and radiation of several land and sea passages through different regions. According to different modes of transportation, it can be divided into land and sea Silk Road. Among them, the land Silk Road can be divided into north and south lines according to the geographical trend. The southern line mainly refers to the vast area from Sichuan to India and even Southeast Asia, and the northern line mainly refers to the area starting from Chang'an, passing through the Hexi Corridor, the Western Regions, and reaching the Mediterranean Sea in the west to ancient Rome.

This paper analyzes the economic growth convergence and spatial effects of countries along the “Belt and Road” by using spatial measurement methods, using panel data, and introducing spatial effects. First, this paper estimates and analyzes the absolute α convergence of the economies of countries along the “Belt and Road.” Hausman's test was used in the selection of fixed-effects and random-effects models. At the same time, after introducing relevant control variables such as population growth rate, capital, labor force, and the proportion of service industry in GDP, etc., this paper estimated and analyzed the convergence of economic conditions α in countries along the “Belt and Road.”

Since the “Belt and Road” initiative is a regional cooperation plan emerging in recent years, there are few empirical analyses on the economic growth convergence and spatial effects of countries along the route. Therefore, this paper analyzes the economic growth convergence and spatial effects of countries along the “Belt and Road” by using spatial measurement methods, using panel data, and introducing spatial effects. Countries are no longer seen as separate, unrelated economies. Rather, it is believed that they have technological spillovers, that the flow of factors of production and trade are correlated, and the closer the distance or the closer they are to each other, the stronger the correlation. First, analyzing whether there is absolute α-convergence and conditional α-convergence in the economies of countries along the “Belt and Road.” Secondly, analyzing the effects of factors of production, population growth, and industrial structure on economic growth. Thirdly, comparing the estimation results of the model without considering the spatial correlation and the model considering the spatial correlation, it shows whether there is a spatial effect in the economic growth of the economies along the “Belt and Road.” Finally, it is explained that the “Belt and Road” initiative is a regional cooperation mechanism that aims to seek common growth and is characterized by openness and inclusiveness. It can bring huge potential markets and supplements of funds and technologies to each member state, and it also requires the joint efforts of countries and regions along the route to improve the initiative.

The “Belt and Road” initiative adheres to the cultural tradition of the ancient Silk Road, and emphasizes openness, tolerance and harmony in cultural concepts. In the field of economic cooperation, we adhere to the concept of common development, benefiting ourselves and others, and “doing what you do not want to do to others,” in order to promote the integration and cooperation of different civilizations, and avoid the dilemma caused by civilizational and economic conflicts. At the same time, it can prevent “cross-infection” between multiple negative factors.

Cooperation focus of the Belt and Road Initiative:

People-to-people bonds are the social foundation of Changyi under the Belt and Road Initiative. Adhering to and carrying forward the concept of peaceful and friendly cooperation on the ancient Silk Road, respecting the wisdom and advantages of different civilizations, it extensively carries out cultural exchanges, academic exchanges, talent training cooperation, and volunteer services. Cooperation in running schools is carried out to strengthen mutual talent training and establish a humane scholarship system, which is conducive to deepening exchanges and mutual trust between countries and civilizations. Strengthening tourism cooperation and simplifying passport application procedures can not only enjoy the social returns brought by tourism resources, but also strengthen non-governmental exchanges and cooperation. It should strengthen cooperation with neighboring regions and countries in the fields of information exchange of infectious disease epidemics, communication of prevention and control technologies, and assistance to improve the ability to respond to public health crises and other natural disasters. At the same time, political parties and non-governmental organizations should be used as bridges to strengthen cooperation and exchanges in politics, humanities, health, and charity.

The proposal and implementation of the “One Belt, One Road” strategy marks China's transformation from the traditional role of bystander and participant to a new role in shaping international cooperation and governance. However, the “One Belt, One Road” strategy is not only a regional development strategy, but also an international cooperation strategy to drive the global economy out of the trough. The number of countries involved, the size of the area, and the amount of capital involved are rare in Chinese history and even in global history. The economic development, ideology and social structure of the countries along the “Belt and Road” construction are complex. How China strengthens cooperation with countries along the route while playing its leading role has become an important factor affecting the implementation of the “One Belt, One Road” strategy and achieving the desired results. Therefore, to ensure that the “Belt and Road” initiative can be effectively carried out, this paper proposes the following policy recommendations:

1. Strengthen cooperation in border areas. According to the center-periphery theory of new economic geography, there are opportunities to invest and build factories in areas far from central cities, which can form new active economic belts. Moreover, the new era has become an important platform for exchanges and trade between countries because the border area is in a state of peaceful development as a whole. In addition, the extensive economic, cultural and social exchanges in the border areas have a good trade foundation and can promote the activation of the cross-border sub-regional market. The “Belt and Road” construction spans a large number of countries along the way, and the border areas are often areas where technology, capital, and human capital are relatively backward. According to the classical growth model, funds have higher returns in these areas. The formation of industrial clusters by investing in factories can well-promote cooperation and trade between countries, and at the same time change the backwardness of this region and improve local welfare. This is in line with the openness, inclusiveness and common prosperity of the Belt and Road construction.
2. China should strengthen cooperation with major countries along the route. The “Belt and Road” construction is a cooperation mechanism that supports the economic rise of China and Asia and injects fresh blood into the global economic recovery. Although proposed and dominated by China, relying solely on China's power is not enough. Therefore, strengthening cooperation with countries and regions along the route, especially the major countries along the route, is related to the smooth implementation of the “Belt and Road” construction. First, in the construction of the “Belt and Road,” China and the United States have economic and political competition in Southeast Asia, South Asia, the Middle East and other regions. Especially in the Middle East and the Gulf region, it has always been a key strategic area for US energy supply and control of the world energy order. China's energy cooperation, expansion of trade, and infrastructure investment in the region will make the United States feel huge challenges and unease, and will easily lead to US countermeasures and interference. In fact, China and the United States have huge potential and prospects for cooperation in energy and resource development and trade along the “Belt and Road.” The Middle East, as the world's “powder keg,” has been in turmoil in the region. Whether it is terrorism or territorial disputes between regions, China and the United States need to work together to maintain regional stability and ensure the normal operation of energy supply and trade order. This is in the interests of both countries and the world. Therefore, China and the US should cooperate in more fields to expand common interests, enhance consensus, and eliminate US resistance to China's “One Road and One Belt” construction.
  Second, China should strengthen cooperation with India. As the two most populous developing countries in the world, China and India have developed rapidly in their economy and energy demand, accounting for the vast majority of the world's energy increment. Therefore, the cooperation between China and India in energy is very important. In terms of energy demand, due to the high dependence of the two countries on oil imports, there is fierce competition between the two countries in seeking energy exporters and finding crude oil origins. Coupled with the non-renewable nature of oil, the characteristics of zero-sum game gradually emerged. As a result, it is very easy to cause tensions in the economic fields of the two countries, and at the same time raise market prices, causing heavy losses to both sides. Therefore, under the framework of the “Belt and Road” construction, the two sides should cooperate with each other in mutual trust, ensure a sound energy market order, play the role of major powers in Asian energy cooperation, and at the same time, strengthen the right to speak together in global energy governance. More importantly, India is a big developing country, and strengthening the infrastructure construction in the “Belt and Road” construction is of great significance to the backwardness of infrastructure. It is also an important foundation on which India can develop rapidly. India should not adopt a passive and passive attitude, but should exert its influence and due responsibilities in the region to speed up the implementation of this strategy and ensure the smooth progress.
3. Actively play the role of the AIIB. The focus of the “Belt and Road” construction is infrastructure construction and investment and trade, and the Asian Infrastructure Construction and Investment Bank (hereinafter referred to as “AIIB”) is a multilateral investment and financing bank initiated by China. At present, it has 57 member countries, covering Europe, Asia, Africa, Oceania and Latin America (except the United States and Japan). The AIIB closely links the interests of its founding member countries. Among them, most countries in Southeast Asia and South Asia directly benefit from the improvement and connectivity of infrastructure in the construction of the “Belt and Road,” while other member countries will win trade benefits due to the economic integration of Europe, Asia and Africa promoted by the construction of the “Belt and Road.” As the largest investor in the AIIB, China should try its best to play the role of the AIIB in reducing investment and financing costs and investment risks. At the same time, the renminbi has joined the SDR, which should promote the renminbi's function as a bilateral and multilateral settlement and reserve currency, and improve the operational efficiency of the Asian capital currency market. In addition, for the AIIB to play its role effectively, it must address the following issues. First of all, it is necessary to do a good job in investment and financing risk assessment, so that capital can play the highest efficiency. Second, because many countries along the “Belt and Road” are relatively backward countries, their credit ratings are relatively low. When issuing loans, it should strengthen supervision and knowledge to avoid losing all investment. Finally, the financial markets and financial systems of countries along the route are still very immature. On the one hand, it is necessary to strengthen the construction of its own financial system. On the other hand, China should learn from the World Bank and the Asian Development Bank in terms of risk assessment and project evaluation, actively learn from developed countries in Europe and the United States, strengthen exchanges, and strive to integrate with the developed Western financial system as soon as possible.

## α convergence model

The specific form of the neoclassical growth model is as follows:


(1) $$\begin{array}{l} W=F\left(P,Q\mu \right) \end{array}$$

W is the output, Q represents the technical level, P and μ represent the capital stock and labor quantity, respectively, and *Qμ* represents the effective labor quantity. The effective output per capita is:


(2) $$\begin{array}{l} f\left(p\right)=F\left(P,Q_{\mu }\right)/Q_{\mu }=F\left(P/Q_{\mu }\right) \end{array}$$

If U represents the total national investment, and saving equals investment when national income reaches equilibrium, then the per capita capital change Δp can be expressed as:


(3) $$\begin{array}{l} \Delta p=\left(U/Q\mu \right)-\left(a+b+c\right)p=\left(jW/Q\mu \right)-\left(a+b+c\right)\\ \;p=jf\left(p\right)-\left(a+b+c\right)p \end{array}$$

Among them, a represents the population growth rate, b represents the technological growth rate, and c represents the depreciation rate.

If t_p_ is used to represent the growth rate of capital per capita, there are:


(4) $$\begin{array}{l} t_{p}=d\left(\Delta p\right)/dp=j\left(df\left(p\right)/dp\right)-\left(a+b+c\right) \end{array}$$

The convergence of per capita income is generally measured by α convergence, which means that countries or regions with lower per capita income have higher economic growth rates than countries or regions with higher per capita income. According to different convergence conditions, it can be divided into absolute α convergence and conditional α convergence.

Absolute α convergence refers to the differences in production conditions (factor endowments, production technology, location factors, knowledge reserves, etc.) and production systems in different economies in the initial stage. Economies in different countries or regions will eventually converge to the same steady state level. The specific manifestation is that the backward economies have a higher growth rate than the advanced economies, which leads to the continuous narrowing of the gap between the income levels of the backward economies and the advanced economies. In the end, the same steady state level will be reached, and the balanced and stable development of the economy in different regions will be realized.

The basis of the alpha convergence model is the Cobb-Douglas production function, which is set as:


(5) $$\begin{array}{l} W_{\mu }={\left(Q_{\mu }K_{\mu }\right)}^{1-\beta }P_{\mu }^{\beta },0<\beta <1 \end{array}$$

Among them is the total output of production, *W*_μ_ is the measurement coefficient of production technology, *Q*_μ_ is the input of labor factors, and *K*_μ_ is the total input of production capital.

This results in output per capita:


(6) $$\begin{array}{l} w_{\mu }=W_{\mu }/Q_{\mu }K_{\mu }=f\left(p_{\mu }\right)=p_{\mu }^{\beta } \end{array}$$

Saving equals investment when the national income is in equilibrium, that is:


(7) $$\begin{array}{l} jW_{\mu }=U_{\mu } \end{array}$$

So the total capital change is:


(8) $$\begin{array}{l} \Delta P=jW_{\mu }-\left(a+b+c\right)P_{\mu } \end{array}$$

The change in capital per capita is:


(9) $$\begin{array}{l} \Delta p_{\mu }=jw_{\mu }-\left(a+b+c\right)p_{\mu }=jp_{\mu }^{\beta }-\left(a+b+c\right)p_{\mu } \end{array}$$

Δ*p*_μ_ = 0, the economy reaches a steady state, and the capital per capita at this time is:


(10) $$\begin{array}{l} p^{\prime }={\left(j/a+b+c\right)}^{1/1-\beta } \end{array}$$

Therefore, the output per capita at equilibrium is:


(11) $$\begin{array}{l} w_{\mu }^{\prime }=W_{\mu }/K_{\mu }=Q_{\mu }w_{\mu }=Q_{\mu }f\left(p_{\mu }^{\prime }\right)=Q_{\mu }p_{\mu }^{\prime } \end{array}$$

Taking the logarithm of both sides gives:


(12) $$\begin{array}{l} \ln w_{\mu }^{\prime }=\ln Q_{\mu }+\ln p_{\mu }^{\prime } \end{array}$$

Generally expressed as:


(13) $$\begin{array}{l} \ln w_{\mu }^{\prime }=\ln w_{0}+b \end{array}$$

Thus, it is further deduced that the economic convergence rate is α:


(14) $$\begin{array}{l} \ln w_{\mu }=\left(1-\alpha \right)\ln w_{\mu -1}+\alpha \ln w_{\mu }^{\prime }+\alpha b\mu \end{array}$$

(15) $$\begin{array}{l} \alpha =\left(1-\beta \right)/\left(a+b+c\right) \end{array}$$

Since the model assumes that a, b, and c are fixed, that is to say, the three variables of convergent economies are consistent, and the general situation is further deduced:


(16) $$\begin{array}{l} \ln w_{n,\mu }=\left(1-\alpha \right)\ln w_{n,\mu -1}+\alpha \ln\left(w_{n,0}^{\prime }+b\mu \right)+\phi_{n,\mu }^{0} \end{array}$$

where n represents a region or country, and $\phi_{n,\mu }^{0}$ is a random item.

Let $\beta_{0}=\alpha \ln\;w_{n,0}^{\prime },\gamma =\left(1-\alpha \right)$, so we get:


(17) $$\begin{array}{l} \ln w_{n,\mu }=\gamma \ln w_{n,\mu -1}+\beta_{0}+\phi_{n,\mu }^{0} \end{array}$$

Thus, the absolute alpha convergence model is obtained:


(18) $$\begin{array}{l} \text{ln(}w_{n,\mu }/w_{n,\mu -1})=\beta_{0}-\alpha \ln w_{n,\mu -1}+\phi_{n,\mu }^{0} \end{array}$$

To get the general expression, let α_0_ = −α , get:


(19) $$\begin{array}{l} \text{ln(}w_{n,\mu }/w_{n,\mu -1})=\beta_{0}+\alpha_{0}\ln w_{n,\mu -1}+\phi_{n,\mu }^{0} \end{array}$$

If α <0, there is absolute α convergence, otherwise, it does not exist. This model is based on the following assumptions: ① The initial state income level is independent of surrounding areas and is exogenous. ② Assume the same population growth rate and technological growth rate in each region. ③ The convergence speed of each region is equal.

Conditional α convergence means that although different countries or regions have similarities in terms of economic structure and market potential, it can be expressed as:


(20) $$\begin{array}{l} \text{ln(}w_{n,\mu }/w_{n,\mu -1})=\beta_{0}+\alpha_{0}\ln w_{n,\mu -1}+\lambda Z_{n,\mu }+\phi_{n,\mu }^{0} \end{array}$$

*Z*_*n*,μ_ is the control variable. If α <0, there is a condition of α convergence, otherwise, it does not exist. The difference between absolute α convergence and conditional α convergence is that the former means that different economies eventually converge to the same steady state level, and there is no gap between different economies. The latter means that different economies are affected by different factors and eventually converge to their own steady state, and the gap between them still exists.

In the absolute α-convergence model of the economy of the 20 countries along the route, both spatial lag autocorrelation and spatial error autocorrelation should be considered in the selection of the panel model. By using the Hausman test, it is found that a fixed effect model should be used, but the spatial correlation in the model may be spatial lag autocorrelation, spatial error autocorrelation, or both. In this paper, the maximum likelihood method is used to estimate the spatial econometric model, and the results are shown in Table 1.

**Table 1:** The results of maximum likelihood estimation of absolute beta convergence of panel data for 20 countries along the route.

**Variables (coefficients)**	**Not considering the spatial phase**	**Spatial lag**	**SARAR**	**Spatial error autocorrelation**
Inyt	−1.043	−1.01427	−1.0476	−1.0245
Splag		1.248	1.273	
Spar			1.452	1.24

The status of medical institutions in a city is shown in Table 2.

**Table 2:** Status of medical institutions in a city.

**Ownership**	**Total number of institutions**	**Total number of beds**	**Registered capital (million yuan)**	**Floor space (square meters)**	**Building area (square meters)**
Non-profit (government-run)	887	14,043	200921.18	1164684.30	1517653.00
For-profit (not government-run)	973	2,944	273109.12	1273283.32	1189953.02
Non-profit (not government-run)	929	2,565	29395.40	456637.43	239253.77
Total	2,789	19,552	503425.73	2894606.05	2946859.79

**Figure 8:**
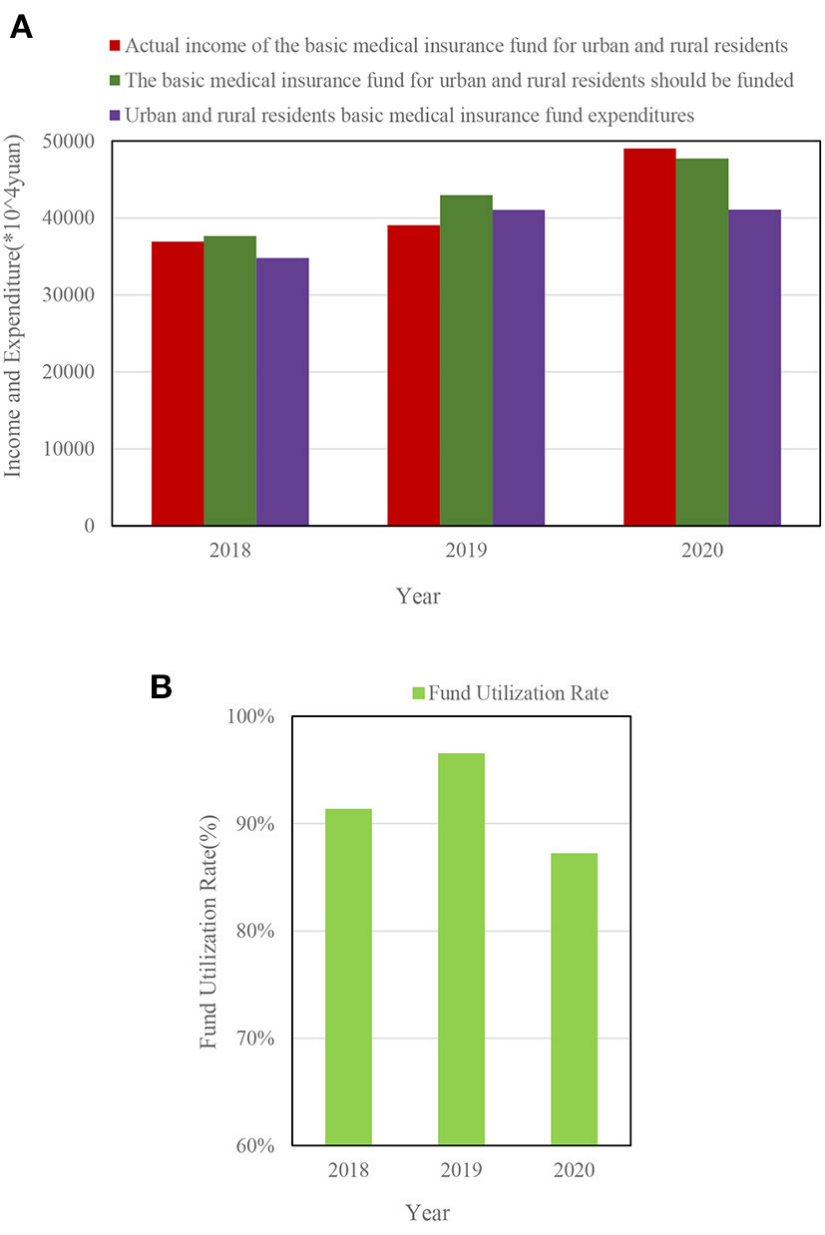
**(A)** Income and expenditure of basic medical insurance fund for urban and rural residents in 2018–2020. **(B)** Utilization rate of medical insurance funds for urban and rural residents in 2018–2020.

Figure 8 shows the income and expenditure of the basic medical insurance fund for urban and rural residents in a certain district.

Figure 8A shows that the income and expenditure of the basic medical insurance fund for urban and rural residents in a certain district increased year by year from 2018 to 2020. Figure 8B shows that the utilization rates of medical insurance funds in 2018–2020 were 91.31, 96.49, and 87.16%.

Table 3 shows the reimbursement benefits of urban and rural residents' medical insurance, urban residents' medical insurance, and new rural cooperative medical care for special slow outpatient services in a city.

**Table 3:** Reimbursement treatment.

	**New agricultural cooperative**	**Urban health insurance**	**Urban and rural medical insurance**
Starting Pay Line	0	0	0
Reimbursement rate (%)	40	60	60
Annual cap line (million yuan)	0.3	5	10.5

Table 3 shows that the new medical insurance system attaches great importance to improving the benefits of special chronic diseases in the process of integrating medical insurance for urban and rural residents.

**Figure 9:**
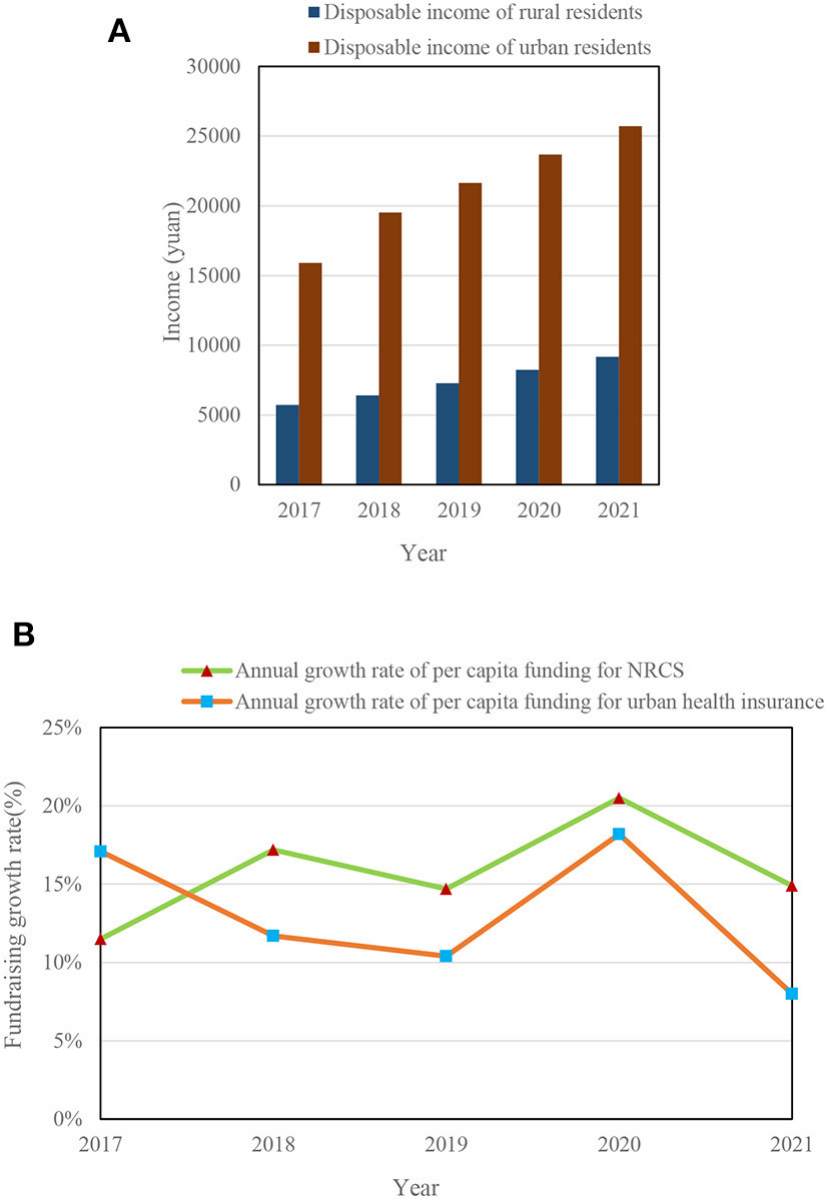
**(A)** Disposable income of rural residents in 2017–2021. **(B)** Average annual growth rate of per capita fundraising in 2017–2021.

Figure 9 shows the per capita disposable income and per capita financing standards of a district from 2017 to 2021.

Figure 9A shows that from 2017 to 2021, the disposable income of rural residents increased from 5,728 to 9,166 yuan, and the disposable income of urban residents increased from 15,914 to 25,720 yuan. Figure 9B shows that the average annual growth rate of per capita fundraising for NCMS is 15.76%, and that for urban medical insurance is 13.08%.

From 2018 to 2021, the gap between the disposable income of urban and rural residents in a certain district and the gap between the insurance financing standards is shown in Table 4.

**Table 4:** 2018–2021 disparity between disposable income of urban and rural residents in a district and disparity in funding standards for participation in insurance.

**Year**	**Urban-rural income gap (yuan)**	**Income gap growth rate (%)**	**Urban and rural health insurance funding gap (yuan)**	**Funding gap reduction rate (%)**
2018	15,149	31.25	49	21.45
2019	16,482	10.45	39	19.14
2020	17,452	8.15	42	10.54
2021	18,946	6.49	0	100

Table 4 shows that the urban-rural income gap widened from 2018 to 2021, and the urban-rural income gap increased from 15,149 to 18,946 yuan from 2018 to 2021.

**Figure 10:**
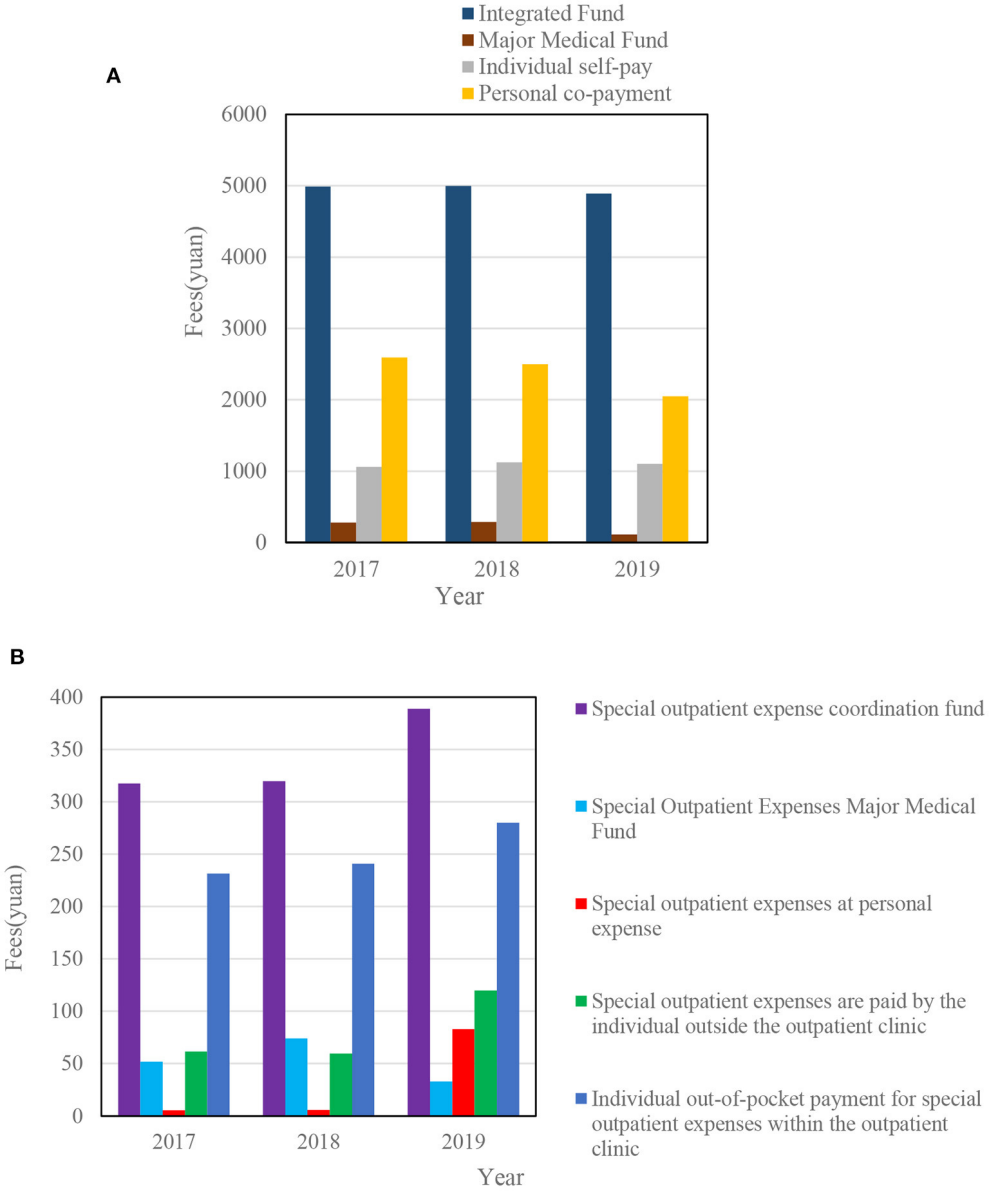
**(A)** Reimbursement of medical expenses for urban and rural residents in 2017–2019. **(B)** Changes in the average out-of-pocket expenses of specialized outpatient clinics for urban and rural residents from 2017 to 2019.

Figure 10 shows the reimbursement of medical expenses for insured urban and rural residents in a district from 2017 to 2019.

Figure 10A shows that the average hospitalization expenses paid by the pooled funds from 2017 to 2019 were 4,986.19, 4,997.34, and 4,888.60 yuan, respectively. Figure 10B shows that from 2018 to 2019, the average out-of-pocket expenses for special outpatient clinics jumped from RMB 5.68 to 82.94.

The basic social medical insurance financing standards for urban and rural residents in a city over the years are shown in Table 5.

**Table 5:** Funding standards of basic social medical insurance for urban and rural residents in a city in previous years.

**Year**	**New agricultural cooperation (yuan)**		**Urban residents' medical insurance (yuan)**	
	**Individual contribution**	**Individual contribution**	**Individual contribution**	**Individual contribution**
2017	70	320	120/50	320
2018	90	380	120	380
2019	120	420	120	420
2020	150	450	150	450
2021	180	450	180	450

Table 5 shows that from 2017 to 2018, the financing standards of urban and rural residents' medical insurance have been increasing rapidly, which greatly increased the income of urban and rural residents' medical insurance pooling fund, which is conducive to increasing the payment capacity of the pooling fund.

**Figure 11:**
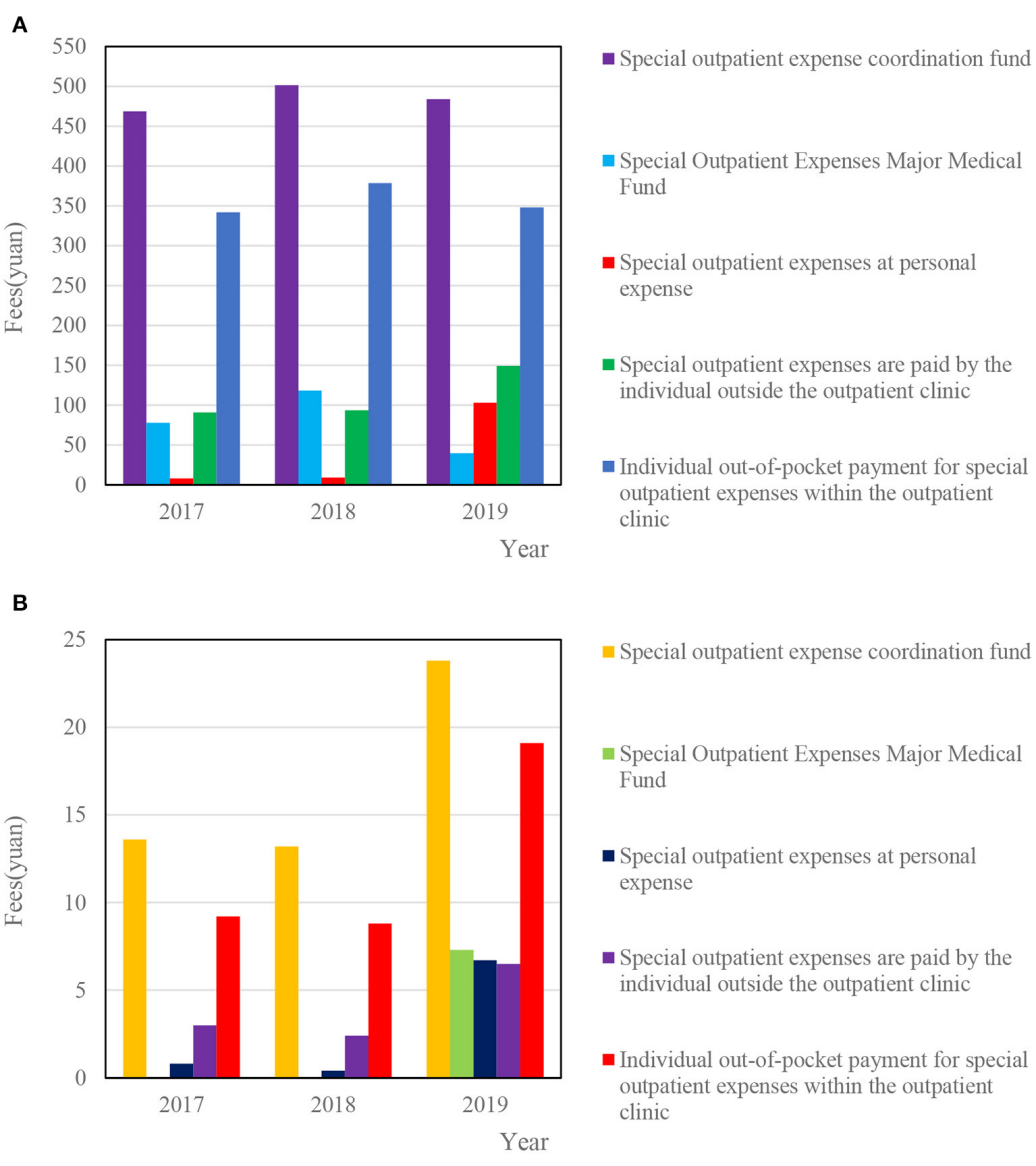
**(A)** Special outpatient coordination fund reimbursement in 2017–2019. **(B)** Individual reimbursement of medical insurance minors for special outpatient expenses in 2017–2019.

Figures 11A,B show the reimbursement of special outpatient expenses for adults and minors under medical insurance for urban and rural residents in a certain district, respectively.

Figure 11A shows that from 2017 to 2019, the reimbursement of the pooled fund for the special outpatient expenses of resident medical insurance adults in a district was 468.5, 501.4, and 483.8 yuan. Figure 11B shows that from 2017 to 2019, the personal reimbursement of resident medical insurance minors in a certain district for special outpatient expenses was 0.8, 0.4, and 6.7 yuan. In addition, in 2019, the average expenses of minors in special outpatient clinics showed strong growth.

## Discussion

The “Belt and Road” is a development path featuring win-win cooperation, mutual respect and mutual trust. It not only meets the needs of China's economic transformation and coping with internal and external pressures, but also meets the needs of countries along the route to accelerate economic growth and strengthen infrastructure. The joint construction of the “Belt and Road” is based on goal coordination and policy communication, with the aim of pursuing common economic growth and prosperity of all countries, oriented to enhance the overall competitiveness of the region, and aimed at narrowing the economic gap between each other. Under the existing framework of bilateral and multilateral, regional and sub-regional cooperation, it would further strengthen the connectivity of countries along the route and improve the level of trade facilitation, so as to inject new impetus and energy into regional economic development.

The prevention and control of the new crown epidemic should strengthen epidemic prevention and control safety education, and pay attention to the epidemic situation in real time. Carry out health education and publicity work for all personnel (managers present and left-behind duty personnel), and popularize epidemic prevention and control knowledge and prevention and control requirements. Wear masks, wash hands frequently, measure body temperature, disinfect frequently, ventilate more, and gather less together on a daily basis, and guide all staff to work together to prevent and control the epidemic. Do not believe rumors, do not spread rumors, and resolutely win the battle against the epidemic.

## Conclusion

Under the circumstance that the “One Belt, One Road” strategy has not been implemented (the natural state), whether in the absolute α-convergence model or in the conditional α-convergence model, the parameter estimation results have shown that there is a significant positive space for the 20 countries along the “Belt and Road” construction, which verified the first hypothesis of this paper. That is, in the natural state, the economic development of various countries has a relationship of mutual promotion rather than mutual competition. Then, after the implementation of the “One Belt, One Road” strategy, by strengthening the construction of interconnected infrastructure and trade facilitation, the connection between countries along the route will be strengthened. And it can promote the dissemination of knowledge and technology. The dissemination of knowledge and technology helps to improve the important factor affecting the production function in the classical growth model, that is, technical means. In this way, if countries along the route can jointly promote infrastructure construction, strengthen mutual connectivity, and give play to their respective comparative advantages. Countries such as Vietnam and India can take advantage of huge demographic dividends, undertake manufacturing transfers from China and other countries, and increase investment in R&D and technology. Then it is expected to catch up with the more developed countries along the route with a higher growth rate. In the conditional α-convergence model, from the perspective of production factor input, capital and population growth are significantly positive for economic growth. It shows that there are still huge demographic dividends in the countries along the “Belt and Road,” and the efficiency of capital investment on economic growth is much higher than that of labor input. From the perspective of industrial structure, the proportion of industrial added value in the added value of workers and peasants has a significant positive effect on the economic growth of countries along the route, indicating that industry plays an important role in promoting economic growth. This research is only aimed at the prevention and control of the COVID-19 and the “One Belt, One Road” economy. In fact, it can also be considered in a wider range, such as the impact of medical innovation policies on employment and so on.

## Data availability statement

The original contributions presented in the study are included in the article/supplementary material, further inquiries can be directed to the corresponding authors.

## Ethics statement

Ethical approval for this study and written informed consent from the participants of the study were not required in accordance with local legislation and national guidelines.

## Author contributions

YC: work concept or design and draft article. ZZ: data collection. WD: make important revisions to the article and approve final article for publication. All authors contributed to the article and approved the submitted version.

## Funding

This work was supported by Major project of Beijing Social Science Foundation Research on Financial Support System Adapting to the Coordinated Development of Strategic Emerging Industries in Beijing-Tianjin-Hebei, No. 20ZDA11.

## Conflict of interest

The authors declare that the research was conducted in the absence of any commercial or financial relationships that could be construed as a potential conflict of interest.

## Publisher's note

All claims expressed in this article are solely those of the authors and do not necessarily represent those of their affiliated organizations, or those of the publisher, the editors and the reviewers. Any product that may be evaluated in this article, or claim that may be made by its manufacturer, is not guaranteed or endorsed by the publisher.
